# Circadian Regulation of Gut Microbial Metabolites in Intestinal Epithelial Homeostasis

**DOI:** 10.3390/metabo16060383

**Published:** 2026-06-01

**Authors:** Miri Park, Sooah Kim, Jeehwan Choe

**Affiliations:** 1 Food Functionality Research Division, Korea Food Research Institute, Wanju 55365, Republic of Korea; pmr@kfri.re.kr; 2 Department of Environment Science & Biotechnology, Jeonju University, Jeonju 55069, Republic of Korea; 3 Department of Livestock, Korea National University of Agriculture and Fisheries, Jeonju 54874, Republic of Korea

**Keywords:** circadian rhythms, gut microbiota, microbial metabolites, intestinal epithelial homeostasis, short-chain fatty acids, secondary bile acids, chrono-nutrition, indole derivatives, intestinal barrier

## Abstract

The gut microbiota produces chemically diverse metabolites whose levels fluctuate depending on the time of day, driven by bidirectional coupling between host intestinal circadian clocks and intrinsic microbial oscillators. Although short-chain fatty acids have received the most attention as microbial circadian effectors, a broad class of metabolites, including secondary bile acids, indole derivatives, and branched-chain fatty acids, engage distinct epithelial receptors and transcriptional programs through mechanisms that are, to varying degrees, subject to circadian regulation. However, the mechanisms by which these metabolite classes collectively regulate barrier integrity, mucosal immune tone, and stem cell-driven renewal, as well as the consequences of their rhythmicity loss under circadian misalignment, have not been systematically reviewed. This review constructs a mechanistic framework linking microbial metabolite rhythmicity to the circadian regulation of intestinal epithelial homeostasis and evaluates dietary and probiotic interventions that modulate this axis as chronobiotic strategies. Convergent mechanisms, unresolved questions, and translational opportunities are identified across in vitro, preclinical, and clinical evidence.

## 1. Introduction

The intestinal epithelium is continuously exposed to chemically diverse gut microbial metabolites whose production oscillates in a pronounced time-of-day-dependent manner. This temporal structure arises from bidirectional coupling between the host intestinal circadian clock and intrinsic oscillators within the gut microbiota, establishing a dynamic metabolic environment that shifts systematically across a 24 h cycle [1,2]. The host circadian clock, governed by a transcription–translation feedback loop involving CLOCK, BMAL1, PER, and CRY proteins, drives rhythmic outputs in the intestinal epithelium, including mucosal immune activity, barrier maintenance, and epithelial cell renewal, which collectively shape the luminal environment in which gut bacteria reside and function [3,4]. Reciprocally, the microbiota and its metabolic products serve as timing cues that entrain peripheral circadian oscillators, establishing a functional axis in which microbial metabolite rhythmicity and intestinal epithelial homeostasis are mutually dependent [5,6].

The functional importance of this axis is underlined by evidence from genetic and germ-free models. Intestinal epithelial cell-specific ablation of the core clock gene *Bmal1* disrupts the rhythmic abundance and metabolic output of the gut microbiota; targeted metabolomics has functionally linked intestinal clock-controlled bacteria to microbial-derived products, including branched-chain fatty acids (BCFAs) and secondary bile acids [7]. Conversely, germ-free (GF) mice, which are devoid of all intestinal microbiota from birth, display attenuated levels of peripheral clock gene expression; colonization with defined microbial consortia partially restores host circadian programs via epigenetic remodeling of the intestinal epithelial chromatin [8,9]. It should be noted that antibiotic-treated (ABX) models, while commonly used as an alternative to GF models, introduce the confound that antibiotic residues and direct drug effects on host epithelial metabolism may independently alter microbial metabolite profiles and circadian outputs, limiting causal interpretation relative to true GF systems [8]. Circadian misalignment arising from shift work, irregular dietary patterns, or clock gene disruption consistently reduces microbial metabolite rhythmicity, impairs intestinal barrier function, and alters mucosal immune tone, collectively linking clock–microbiota decoupling to gastrointestinal pathology [10,11].

Despite this mechanistic foundation, the existing literature has been disproportionately focused on SCFAs as the primary microbial mediators of circadian entrainment, as exemplified by extensive characterization of butyrate-mediated HDAC inhibition at clock gene promoters and SCFA-driven phase resetting in murine enteroid models [8,9], whereas equivalent circadian evidence for secondary bile acids, indole derivatives, and BCFAs remains sparse. The gut microbiota produces a substantially broad repertoire of bioactive metabolites that engage distinct epithelial receptors and transcriptional programs in a time-dependent manner, including secondary bile acids, indole derivatives, and BCFAs [12,13,14]. However, the mechanisms by which these metabolite classes collectively regulate barrier integrity, mucosal immune tone, and stem cell-driven renewal, as well as the consequences of their rhythmicity loss under circadian disruption, have not been comprehensively analyzed within a unified mechanistic framework.

This review addresses this gap by constructing a metabolite-centric mechanistic framework for the circadian governance of intestinal epithelial homeostasis. For each of four major microbial metabolite classes, we examine the mechanisms by which host and microbial circadian systems co-regulate time-dependent metabolite production, their time-gated effects on intestinal epithelial function, and the downstream consequences of rhythmicity loss. We further evaluate dietary and probiotic interventions as chronobiotic strategies targeting the gut microbiota–epithelium circadian rhythm axis and identify convergent principles, unresolved questions, and translational opportunities for the design of temporally precise nutritional strategies in gut health. A comparative overview of the four metabolite classes across production, signaling, immune, and chronobiotic dimensions is provided in Table 1.

## 2. The Host–Microbiota Circadian Rhythm Axis: Framework and Principles

### 2.1. Bidirectional Coupling Between Host Intestinal Clock and Microbial Oscillators

The host circadian system operates through a hierarchical architecture in which the master pacemaker in the suprachiasmatic nucleus (SCN) entrains peripheral clocks distributed across metabolically active tissues, including the intestinal epithelium, liver, and adipose tissue, via hormonal, neural, and feeding-fasting signals [3,12]. In the intestinal epithelium, the peripheral clock is driven by an autonomous transcription–translation feedback loop in which the CLOCK-BMAL1 heterodimer activates the transcription of *Per* and *Cry*, whose protein products accumulate and repress their own transcription over an approximately 24 h cycle. Moreover, an auxiliary loop involving REV-ERBα and RORα competitively regulates *Bmal1* expression, conferring robustness and tissue specificity to the oscillation [3,4]. Through clock-controlled genes, this molecular machinery drives rhythmic outputs in the intestinal epithelium, encompassing barrier permeability, mucus secretion, antimicrobial peptide production, and innate immune tone, collectively structuring the temporal niche that luminal bacteria inhabit [14,28,52].

The host clock exerts top-down control over the gut microbiota via multiple physiological intermediaries. Feeding-fasting cycles, which are regulated by the SCN, generate rhythmic nutrient availability that entrains microbial growth and fermentative activity. The rhythmic secretion of bile acids under hepatic clock control selectively favors microbial taxa capable of bile acid transformation at specific times of day. Antimicrobial peptides such as REG3γ and lipocalin-2, whose expression oscillates via BMAL1-dependent and microbiota-stimulated STAT3 signaling [53], further shape the circadian landscape of mucosal microbial colonization [28,53]. The causal role of the intestinal epithelial clock in driving microbial rhythmicity has been directly demonstrated: intestinal epithelial cell-specific ablation of *Bmal1* disrupts diurnal oscillations in microbial community composition and metabolic output independently of the central clock; targeted metabolomics has functionally linked intestinal clock-controlled bacteria to the rhythmic production of BCFAs and secondary bile acids [7].

**Figure 1:**
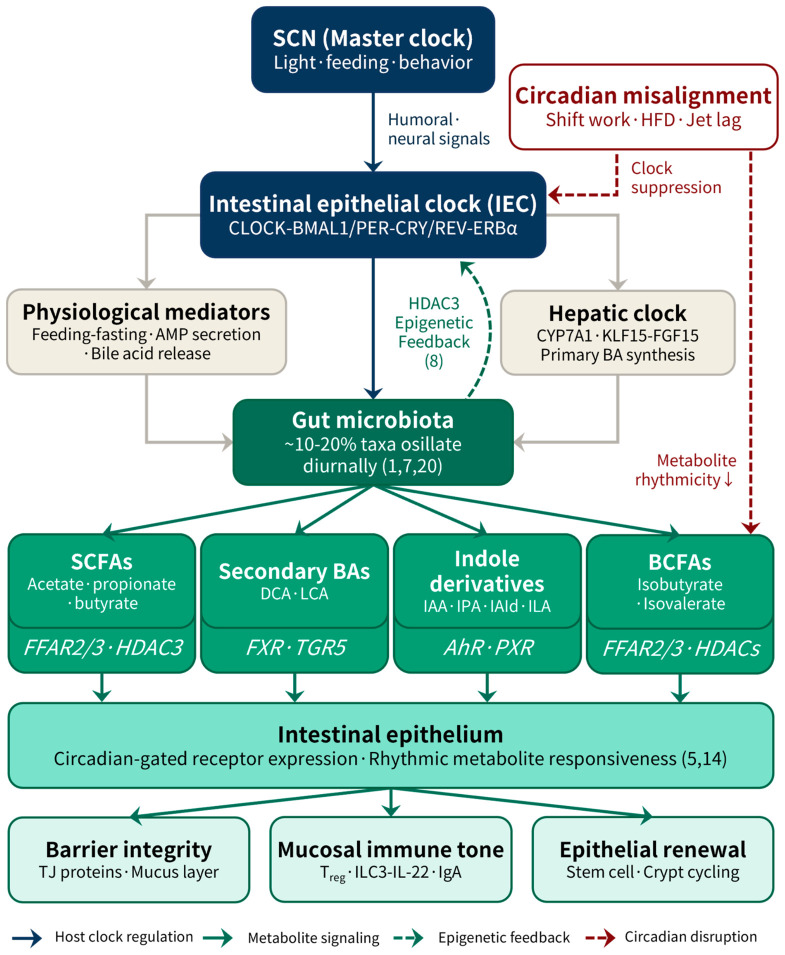
Bidirectional coupling of the host–microbiota circadian rhythm axis and microbial metabolite rhythmicity. The suprachiasmatic nucleus (SCN) entrains the intestinal epithelial clock (IEC) via humoral and neural signals, coordinating feeding-fasting rhythms, antimicrobial peptide secretion, and hepatic bile acid synthesis. These host outputs entrain the gut microbiota, of which approximately 10–20% of taxa oscillate diurnally, whereas microbiota-derived signals reciprocally reinforce host clock amplitude via histone deacetylase 3 (HDAC3)-mediated epigenetic remodeling. Four metabolite classes produced by oscillating taxa signal to the intestinal epithelium via circadian-gated receptors, with rhythmicity-dependent epithelial evidence strongest for SCFAs and secondary bile acids. Notably, the IEC functions not only as a recipient of SCN-derived entrainment but also as a self-sustained peripheral oscillator that independently regulates microbial metabolite production rhythmicity through epithelial clock output. Solid navy arrows, host clock regulation; solid teal arrows, metabolite signaling; dashed teal arrows, epigenetic feedback; dashed red arrows, circadian disruption; gray solid arrows, feedback regulatory relationships; ↓, decreased. Abbreviations: IEC, intestinal epithelial cell.

Reciprocally, the microbiota programs host circadian gene expression via epigenetic and receptor-mediated mechanisms [30]. The gut microbiota drives rhythmic histone deacetylase 3 (HDAC3) activity in intestinal epithelial cells, coordinating the diurnal acetylation of metabolic gene promoters and synchronizing lipid uptake with the feeding cycle [4,8]. While HDAC3 is the best-characterized circadian effector in this context, other HDAC family members, including HDAC1 and HDAC2, may also contribute to clock-dependent chromatin remodeling in the intestinal epithelium, though their specific circadian roles remain to be elucidated. Germ-free mice display globally attenuated circadian amplitude [54], with decreased levels of *Bmal1* and *Cry1* transcripts and increased levels of *Per1* and *Per2* transcripts relative to conventionally colonized animals. These alterations are partially corrected by colonization with defined microbial consortia or by the administration of specific microbial metabolites [9]. Microbiota-derived signals further regulate the circadian expression of NFIL3 (E4BP4), a clock-adjacent transcription factor that governs lipid uptake and body composition, in intestinal epithelial cells, establishing a microbiota–epigenetic circuit that drives broad circadian transcriptional programs in the gut epithelium [4,55]. The hierarchical architecture of this host–microbiota circadian rhythm axis and its downstream metabolite signaling pathways are illustrated in Figure 1.

### 2.2. Circadian Regulation of Microbial Metabolite Production: General Principles

The temporal organization of microbial metabolite production is a direct functional consequence of the host–microbiota circadian rhythm coupling described above. Approximately 10–20% of the gut microbial taxa in mice and humans exhibit statistically significant diurnal oscillations in relative abundance; these oscillations are accompanied by corresponding fluctuations in their metabolic outputs [1,51]. The rhythmic substrate availability generated by host feeding-fasting cycles provides the primary extrinsic entrainment cue, whereas the host intestinal clock provides an intrinsic gating mechanism by which epithelial physiological rhythms shape the microbial metabolic environment [7,28]. Consequently, microbial metabolite concentrations in the intestinal lumen, portal circulation, and systemic plasma exhibit pronounced time-of-day variations, with distinct metabolite classes peaking at different circadian phases according to the metabolic specialization of the taxa producing them [2,51].

This temporal organization is not incidental. The time-of-day-dependent production of microbial metabolites creates a dynamic signaling environment to which the intestinal epithelium responds via circadian-gated receptor expression. Epithelial receptors transducing signals from SCFAs, bile acids, and indole derivatives show varying degrees of circadian regulation. FXR and TGR5 exhibit well-characterized diurnal expression patterns in intestinal epithelial cells [13,56], whereas circadian gating of FFAR2/3 and AhR has been demonstrated in specific contexts but requires further systematic characterization [5,14]. This receptor-level gating amplifies the functional consequences of metabolite rhythmicity: a given metabolite concentration encountered at peak receptor expression will elicit a substantially different epithelial response compared with the same concentration encountered at trough expression [57]. Therefore, the integrity of microbial metabolite rhythmicity is a reflection of microbial ecology and a functional prerequisite for the temporal precision of host–microbiota communication at the intestinal epithelial interface. This rhythmicity is particularly vulnerable to disruption under pathological conditions such as obesity, chronic intestinal inflammation, and colitis, in which circadian clock amplitude is attenuated and microbial community oscillations are blunted, amplifying the downstream consequences for epithelial homeostasis [10,11].

### 2.3. Consequences of Circadian Misalignment for Microbial Metabolite Rhythmicity

Circadian misalignment, which arises from the genetic disruption of clock gene function, environmental perturbations such as shift work and artificial light exposure, or chronic dietary patterns inconsistent with circadian physiology, consistently attenuates the amplitude and coherence of microbial metabolite rhythmicity [58]. In *Bmal1*-deficient mice, the diurnal oscillations of SCFA levels in cecal contents are abolished; secondary bile acid rhythmicity is concurrently disrupted, demonstrating that host clock integrity is required to sustain normal metabolite rhythmicity [7,15]. Environmentally induced circadian disruption via chronic light-dark cycle shifting or simulated jet lag similarly reduces the proportion of rhythmically oscillating microbial taxa and dampens SCFA and bile acid oscillations in the intestinal lumen [7,59]. Clinical evidence from individuals with shift work disorder corroborates these findings: multi-omics profiling reveals altered microbial metabolite profiles and impaired barrier function, with changes in the levels of specific metabolites, including dicarboxylic fatty acids, which are linked to mucosal immune activation [11,58,60].

The downstream consequences of metabolite rhythmicity loss for intestinal epithelial homeostasis are multifaceted and extend across barrier, immune, and regenerative functions. Attenuated SCFA rhythmicity reduces the temporal precision of HDAC inhibition at clock gene promoters in the epithelium, creating a self-reinforcing cycle in which metabolite arrhythmicity further dampens host clock amplitude [8,9,36,43]. Loss of secondary bile acid rhythmicity disrupts the diurnal entrainment of FXR and TGR5 signaling in the epithelium, impairing mucosal immune tone and barrier maintenance [13]. At the molecular level, cross-talk between these pathways occurs through FXR-mediated suppression of SCFA-producing microbial taxa via antimicrobial peptide induction, and conversely, butyrate-driven HDAC inhibition modulates FXR target gene expression, creating bidirectional regulatory coupling between SCFA and bile acid circadian signaling [43]. Moreover, the reduced amplitude of indole derivative production attenuates AhR-mediated epithelial barrier gene expression and antimicrobial peptide secretion in a time-dependent manner [14]. These converging consequences establish metabolite rhythmicity loss as a mechanistically coherent upstream driver of intestinal epithelial dysfunction associated with circadian misalignment, providing a conceptual foundation for the metabolite class-specific analyses presented in subsequent sections.

## 3. Major Classes of Rhythmic Microbial Metabolites

### 3.1. SCFAs

SCFAs, primarily acetate, propionate, and butyrate, are produced via the anaerobic microbial fermentation of dietary fibers in the large intestine; they represent the most extensively characterized class of microbial metabolites in circadian biology. Their luminal concentrations exhibit robust diurnal oscillations, with acetate and butyrate levels peaking during the active phase of the host and correlating with the temporal pattern of fiber intake and microbial fermentative activity [16,29,61]. In mice fed ad libitum, significant rhythmicity is detected in the total cecal SCFA content across a 24 h cycle, with peak concentrations coinciding with the onset of the dark (active) phase and low cecal pH reflecting peak fermentative output at these time points [16]. These oscillations are driven by the convergence of feeding-fasting cycles, which determine substrate availability for fermentation, and the host intestinal circadian clock, which shapes the composition and spatial distribution of SCFA-producing taxa via the rhythmic control of luminal physicochemistry and mucosal immune outputs [1,7,43,62,63].

Contribution of the host clock to SCFA rhythmicity is evidenced by the loss of fecal SCFA oscillations in *Bmal1*-deficient mice even when feeding behavior remains intact [15,22,23,64,65]. Conversely, the SCFA production rhythm is substantially restored by time-restricted feeding (TRF) in clock-disrupted models, indicating that feeding timing can partially compensate for the loss of host clock-driven entrainment of SCFA-producing taxa [45]. At the level of microbial taxa, members of *Clostridiales*, *Lachnospiraceae*, and *Ruminococcaceae*, the primary butyrate producers in the mammalian colon, exhibit circadian oscillations in abundance that parallel the diurnal pattern of butyrate production, underlining the correspondence between microbial community rhythmicity and metabolite output rhythmicity [1,12].

### 3.2. Secondary Bile Acids

Secondary bile acids are generated via the microbial enzymatic transformation of host-derived primary bile acids (cholic acid and chenodeoxycholic acid) in the colon, primarily via dehydroxylation, deconjugation, and oxidation reactions carried out by *Clostridiales*, *Bacteroides*, and other taxa expressing bile salt hydrolase (BSH) and 7α-dehydroxylase activities [19,20,38,48]. The resulting secondary bile acids, with deoxycholic acid (DCA) and lithocholic acid (LCA) being the most abundant, exhibit diurnal oscillations in luminal and systemic concentrations that are driven by the convergence of hepatic clock-regulated primary bile acid synthesis and circadian activity of bile acid-transforming microbial taxa [5,17]. In humans, robust daily rhythms in the circulating bile acid pool have been demonstrated under entrained conditions, with conjugated bile acids exhibiting significant temporal cross-correlations with plasma lipid species. Critically, these rhythms are substantially attenuated following sleep deprivation and largely lost when environmental timing cues are held constant, indicating that external zeitgebers are the dominant entrainment cues for bile acid rhythmicity in humans [17,24,66]. At the microbial level, intestinal clock ablation disrupts the rhythmic abundance of bile acid-transforming taxa and concurrently attenuates secondary bile acid oscillations in cecal contents, functionally linking the host intestinal clock to the diurnal output of secondary bile acid metabolism [7].

Secondary bile acids signal to the intestinal epithelium via two principal receptor systems. FXR, a nuclear receptor expressed at high levels in ileal enterocytes, is activated by primary and secondary bile acids and regulates a transcriptional program encompassing bile acid transport, innate immune tone, and intestinal barrier gene expression [26,27,67]. FXR activation induces FGF19 secretion from enterocytes; FGF19 signals to the hepatic FGFR4 receptor to suppress CYP7A1-mediated primary bile acid synthesis in a feedback loop that is subject to circadian regulation. This mechanism is attributable to hepatic CYP7A1 expression oscillating under the control of the CLOCK–BMAL1 complex via KLF15–FGF15 signaling [19]. TGR5, a membrane-bound G protein-coupled receptor activated preferentially by secondary bile acids, including LCA and DCA, is expressed in intestinal epithelial cells and enteroendocrine L-cells, where it stimulates MLCK signaling to reinforce barrier function and induces GLP-1 secretion via the cAMP/PKA pathway [26]. TGR5 activation additionally promotes intestinal epithelial cell proliferation by advancing cell cycle progression via Cyclin D1 upregulation, supporting epithelial renewal in a dose- and timing-dependent manner [27].

The identification of a direct molecular link between a specific secondary bile acid and the core circadian clock machinery is a mechanistically significant recent development. LCA lengthens the circadian period in human colonic cells by modulating the CK1δ/ε-PP1 feedback loop and stabilizing the core clock protein CRY2, revealing a pathway via which microbially produced bile acid metabolites directly influence the kinetics of the circadian oscillator in intestinal epithelial cells [13,44]. This finding establishes secondary bile acids as downstream effectors of circadian-regulated processes and direct modulators of clock protein stability, adding a reciprocal regulatory dimension to the host–microbiota circadian rhythm axis [68]. Consistent with this finding, circadian disruption-associated changes in secondary bile acid composition, including reduced DCA and LCA levels observed in *Bmal1*-deficient colitis models, are accompanied by dysbiosis and impaired mucosal immune tone, connecting secondary bile acid rhythmicity loss to intestinal inflammatory susceptibility [12,66].

### 3.3. Indole Derivatives

Indole derivatives are produced via the microbial catabolism of dietary tryptophan in the intestinal lumen, with principal metabolites such as indole-3-acetic acid (IAA), indole-3-propionic acid (IPA), indole-3-aldehyde (IAld), indole-3-lactic acid (ILA), and indole itself. These metabolites are generated by various microbial taxa, including *Lactobacillus*, *Clostridium*, *Bacteroides*, and *Bifidobacterium* species expressing tryptophanase and related enzymes [42,69,70]. The production of these metabolites is subject to circadian gating via two convergent mechanisms: the temporal availability of dietary tryptophan following host feeding cycles and circadian oscillation of tryptophan-metabolizing bacterial taxa whose abundance varies with the host feeding-fasting rhythm [1,6,18,21]. Consequently, luminal indole derivative concentrations exhibit time-of-day variation that is coupled to the broad pattern of microbial metabolic activity, with production peaking during and following the active feeding phase of the host [47].

Indole derivatives signal to the intestinal epithelium primarily via the AhR, which is a ligand-activated transcription factor expressed in intestinal epithelial cells, intraepithelial lymphocytes, and innate lymphoid cells [32,69]. AhR activation by IAA, IPA, IAld, and ILA upregulates tight junction protein expression and distribution in epithelial cells, reduces paracellular permeability, and suppresses pro-inflammatory NF-κB signaling, collectively reinforcing intestinal barrier integrity [32,34,49,71]. AhR activation in group 3 innate lymphoid cells (ILC3s) by microbially derived indole derivatives induces IL-22 production, which stimulates epithelial proliferation, mucus secretion, and antimicrobial peptide expression, constituting an indole–AhR–IL-22 axis that coordinates mucosal immune defense with epithelial renewal [14,42,71]. ILC3s are also directly modulated by SCFAs through FFAR2/3 signaling, which promotes IL-22 production independently of AhR, suggesting that indole and SCFA pathways may act in a complementary or additive manner to sustain mucosal immune tone [36]. IPA additionally increases transepithelial resistance and upregulates MUC2 and MUC4 expression via AhR and pregnane X receptor (PXR) pathways, reinforcing the mucus barrier in a manner complementary to the tight junction effects of other indole metabolites [33,35,69].

**Figure 2:**
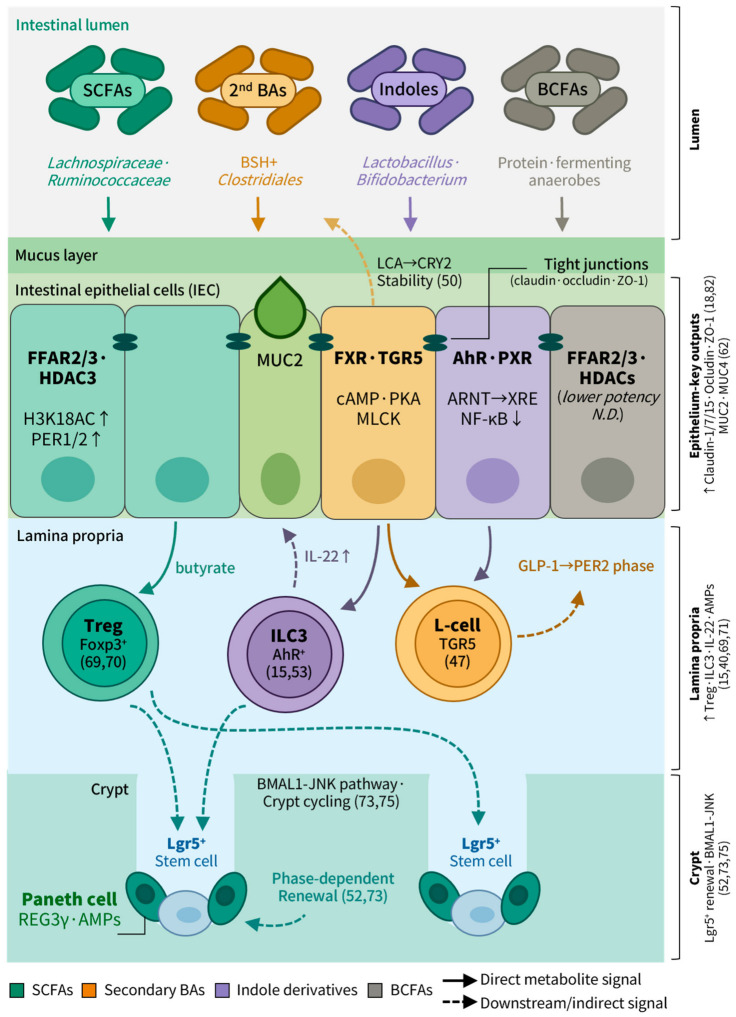
Microbial metabolite signaling at the intestinal epithelial interface: receptor mechanisms, immune cell interactions, and crypt renewal. A cross-section of the intestinal wall illustrates zone-specific signaling by four gut microbial metabolite classes: short-chain fatty acids (SCFAs) activate FFAR2/3 and histone deacetylase 3 (HDAC3) to drive histone acetylation and *Per1*/*2* expression; secondary bile acids signal via farnesoid X receptor (FXR) and Takeda G protein-coupled receptor 5 (TGR5) to reinforce tight junction integrity, with lithocholic acid (LCA) additionally stabilizing CRY2 to lengthen the circadian period; indole derivatives activate aryl hydrocarbon receptor (AhR) and pregnane X receptor (PXR) to upregulate tight junction proteins and suppress NF-κB; and branched-chain fatty acids (BCFAs) are included as an emerging candidate class whose direct epithelial mechanisms remain to be established. In the lamina propria, butyrate promotes Foxp3+ Treg differentiation; indole metabolites drive group 3 innate lymphoid cell (ILC3)-dependent IL-22 production, supporting epithelial renewal; and secondary bile acids stimulate TGR5-mediated GLP-1 release from L-cells to advance to the PER2 phase. At the crypt base, BMAL1-JNK activity gates Lgr5+ stem cell proliferation timing. The figure illustrates both directions of the metabolite–clock relationship: the circadian clock gates epithelial receptor expression and metabolite responsiveness (top-down), while metabolites such as butyrate (via HDAC inhibition) and LCA (via CRY2 stabilization) directly feedback to modulate clock gene expression and period (bottom-up). Circadian misalignment convergently reduces HDAC3, FXR/TGR5, and AhR signaling, initiating a self-reinforcing permeability loop. Solid arrows, direct metabolite signals; dashed arrows, downstream or indirect signals; ↑, increased or upregulated. ↓, decreased. Abbreviations: BSH, bile salt hydrolase; IEC, intestinal epithelial cell; LPS, lipopolysaccharide; MLCK, myosin light chain kinase; Treg, regulatory T cell.

The circadian dimension of indole derivative signaling is conferred by the time-gated expression of AhR in the intestinal epithelium, which oscillates under circadian control, creating windows of differential epithelial responsiveness to indole signals across a 24 h cycle [14]. Circadian disruption, particularly under conditions of tryptophan-metabolizing microbial dysbiosis, attenuates luminal indole derivative availability and reduces AhR-mediated barrier gene expression, contributing to increased epithelial permeability. The indole–AhR axis is mechanistically distinct from the SCFA and bile acid disruption pathways as it operates via transcriptional co-activation, whereby ligand-activated AhR dimerizes with the aryl hydrocarbon receptor nuclear translocator (ARNT) to drive xenobiotic response element (XRE)-dependent gene expression, rather than via epigenetic remodeling or nuclear receptor signaling. Beyond intestinal epithelial effects, the indole–AhR axis extends to hepatic metabolism, where IPA and IAA regulate AhR-dependent lipid and glucose homeostasis, and to neuroimmune interactions, where tryptophan-derived indoles modulate microglial activation and serotonin biosynthesis via gut–brain signaling [42,69]. These cross-system effects underscore the broader physiological relevance of indole derivative rhythmicity beyond the intestinal epithelium. Together, these findings illustrate the complementary and non-redundant nature of the multiple metabolite–clock–epithelium regulatory circuits, as depicted in Figure 2.

### 3.4. Branched-Chain Fatty Acids

BCFAs, principally isobutyrate, isovalerate, and 2-methylbutyrate, are produced via microbial fermentation of branched-chain amino acids rather than dietary fibers, mechanistically distinguishing them from SCFAs [7]. Their relevance to circadian intestinal regulation derives primarily from targeted metabolomics in intestinal epithelial clock gene-deficient mice, in which BCFAs were among the microbial products most significantly linked to *Bmal1*-controlled bacteria, with rhythmic production disrupted by intestinal epithelial *Bmal1* deletion [7]. This finding positions BCFAs as functionally downstream outputs of the host intestinal clock operating through microbial intermediaries rather than as independent circadian effectors. Whether this rhythmicity primarily reflects clock-driven shifts in the composition of BCAA-fermenting taxa or oscillations in the metabolic flux of resident communities remains unresolved and represents a key question for future investigation [7]. Direct evidence for BCFA-specific effects on barrier integrity, mucosal immunity, or epithelial renewal remains limited and largely inferred from structural analogy with SCFAs. BCFAs therefore represent an emerging candidate class whose rhythmic production may serve as a functional readout of host–microbiota circadian decoupling, pending systematic investigation with BCFA-specific experimental models.

Current evidence for the direct effects of BCFAs on intestinal epithelial function remains more limited than that for SCFAs, bile acids, or indole derivatives. BCFAs share a partial mechanistic overlap with SCFAs via HDAC inhibition, although with an inferior potency. Moreover, they activate some of the same free fatty acid receptors, suggesting that their epithelial effects may be qualitatively similar but quantitatively attenuated relative to SCFAs [25,29]. Their diurnal production pattern, which is tightly linked to the host intestinal clock through microbial community dynamics rather than through direct substrate availability, makes them a particularly informative marker of intestinal clock integrity. Hence, the loss of BCFA rhythmicity in clock-disrupted models serves as a functional readout of host–microbiota circadian rhythm decoupling. The characterization of BCFA-specific epithelial effects and their contribution to barrier, immune, and regenerative functions independent of SCFA co-production remains unanswered and warrants systematic investigation. Further studies should employ BCFA-specific receptor knockout models and time-restricted BCAA supplementation to further evaluate their SCFA-independent effects.

## 4. Consequences of Metabolite Rhythmicity Loss on Intestinal Epithelial Homeostasis

### 4.1. Convergent Disruption of Intestinal Barrier Integrity

Increased intestinal epithelial permeability is the most consistently documented consequence of microbial metabolite rhythmicity loss; it arises via the convergent attenuation of multiple barrier-reinforcing metabolite signals. Under conditions of circadian misalignment, the reduction in SCFA and secondary bile acid rhythmicity removes well-characterized barrier-protective inputs to the epithelium; the contribution of attenuated indole derivative rhythmicity to this convergent effect is mechanistically plausible but less directly established. Attenuated SCFA rhythmicity reduces HDAC3-dependent histone acetylation at tight junction gene promoters, impairing the circadian amplitude of *Cldn7*, *Cldn15*, and *Ocln* expression [30,31,43]. Concurrent loss of secondary bile acid oscillations disrupts FXR-mediated barrier gene transcription and TGR5-dependent MLCK signaling in epithelial cells [26,27]. Reduced indole derivative availability attenuates the AhR-mediated upregulation of tight junction proteins and suppresses IL-22-driven epithelial renewal from ILC3s [28,50]. The net effect of this multi-pathway convergence is a barrier vulnerability that exceeds what any single metabolite deficit can produce, explaining the pronounced increase in intestinal permeability observed in models of comprehensive circadian disruption [72,73]. This additive effect arises because each pathway targets distinct molecular components of barrier maintenance: HDAC3-dependent chromatin remodeling governs tight junction gene transcription, FXR/TGR5 signaling regulates cytoskeletal contractility via MLCK, and AhR activation controls barrier gene expression and IL-22-driven epithelial renewal, such that loss of all three simultaneously removes complementary and non-redundant layers of protection.

Clinical evidence from human shift workers confirms this convergent pattern. Metabolomic profiling of individuals with shift work disorder has revealed altered intestinal metabolite signatures, including increased levels of dicarboxylic fatty acids and dysregulated mucosal microbial composition, with concurrent impairment of the intestinal mucus barrier and activation of mucosal B cell responses [60]. These findings indicate that metabolite rhythmicity loss translates into tangible mucosal pathology in humans and is not confined to preclinical models. The self-reinforcing nature of this disruption is mechanistically important: increased intestinal permeability permits microbial translocation and endotoxin entry into the portal circulation, which activates systemic inflammatory signaling that, in turn, suppresses clock gene expression in intestinal epithelial cells, further attenuating metabolite rhythmicity and amplifying barrier dysfunction in a pathological positive feedback loop [60,74].

### 4.2. Dysregulation of Mucosal Immune Tone

Microbial metabolite rhythmicity is an essential input to the temporal organization of mucosal immunity. The diurnal oscillation of IgA secretion, antimicrobial peptide production, and innate lymphoid cell activity in the intestinal mucosa is coordinated by the convergent actions of SCFA-, bile acid-, and indole-derived signals on immune cell populations and epithelial secretory function [30,54]. Loss of metabolite rhythmicity disrupts this coordination, shifting mucosal immune tone toward a sustained pro-inflammatory state that lacks the diurnal variation required for appropriate pathogen response and tolerance of commensal bacteria [61]. In *Bmal1*-deficient intestinal epithelial clock models, microbiota transfer into germ-free recipients demonstrates that arrhythmic microbial communities, which produce attenuated and arrhythmic metabolite outputs, are sufficient to increase lymphoid organ weights and suppress immune cell recruitment patterns in otherwise healthy hosts [7]. This finding establishes that metabolite rhythmicity, rather than total metabolite quantity alone, is a functionally relevant parameter for mucosal immune calibration.

The mechanistic basis of this immune dysregulation involves multiple convergent pathways. Attenuated SCFA rhythmicity reduces the butyrate-mediated induction of regulatory T cell differentiation in the intestinal lamina propria, impairing the circadian regulation of mucosal immune tolerance [37,75,76]. Reduced secondary bile acid rhythmicity attenuates the FXR-mediated suppression of intestinal inflammatory signaling and diminishes the diurnal entrainment of bile acid-sensitive immune cell populations [26,27]. Further, the loss of indole derivative oscillations reduces AhR activation in ILC3s, attenuating rhythmic IL-22 production and the temporal coordination of mucosal antimicrobial defense [50]. Collectively, these converging deficits produce a mucosal immune landscape characterized by elevated baseline inflammation, reduced pathogen resistance amplitude, and impaired tolerance mechanisms, consistent with the inflammatory phenotypes observed in models of clock gene disruption and circadian misalignment [39,42].

### 4.3. Impairment of Stem Cell-Driven Epithelial Renewal

Intestinal epithelial renewal, driven by *Lgr5*+ stem cells in the crypts of Lieberkühn, is subject to circadian gating via the BMAL1-dependent regulation of JNK stress signaling, Wnt pathway activity, and crypt cell proliferation timing [40,69]. Microbial metabolites, particularly SCFAs and bile acids, modulate the temporal pattern of stem cell activity and differentiation through their effects on epigenetic programming and receptor-mediated signaling in the crypt compartment. Under conditions of metabolite rhythmicity loss, the circadian gating of stem cell-driven renewal is disrupted, affecting the rate and precision of epithelial turnover. *Bmal1* deletion in the intestinal epithelium impairs rhythmic proliferation during homeostatic conditions and regeneration following injury, whereas the loss of BMAL1-dependent JNK pathway timing disrupts the coordination of inflammatory cytokine production with proliferative responses in the crypt epithelium [40].

The interplay between metabolite rhythmicity and epithelial renewal extends to the mucus layer. *Bmal1* deficiency in intestinal epithelial cells dampens *Muc2* expression in a time-dependent manner, attenuating the circadian renewal of the mucus barrier, which depends on rhythmic goblet cell secretory activity [35]. The butyrate-mediated suppression of crypt stem cell proliferation in favor of differentiation toward absorptive colonocytes is phase-dependent, such that arrhythmic butyrate delivery impairs the temporal precision of this lineage allocation decision [40,69,77]. Collectively, the compounding effect of increased barrier permeability, immune tone dysregulation, and impaired epithelial renewal under conditions of metabolite rhythmicity loss constitutes a mechanistically coherent and clinically relevant pathway from circadian misalignment to intestinal epithelial dysfunction, providing a conceptual foundation for chronobiotic intervention strategies.

## 5. Chronobiotic Strategies: Dietary and Probiotic Interventions

### 5.1. Time-Restricted Feeding

TRF, the confinement of caloric intake to a defined daily window aligned with the active phase of the host, represents the most robustly supported non-pharmacological strategy for restoring microbial metabolite rhythmicity [41,78]. By reimposing rhythmic substrate availability on fermentative and bile acid-transforming microbial communities, TRF restores the diurnal oscillations of SCFA, secondary bile acid, and indole precursor levels without requiring dietary composition changes, demonstrating that feeding timing is a dominant entrainment cue for microbial metabolite rhythmicity independent of dietary content [46,79]. In *IL-10*-deficient colitis models, TRF during the active phase restores SCFA and secondary bile acid rhythmicity, ameliorates intestinal inflammation, and enhances epithelial regeneration; however, these protective effects are abolished in mice with intestinal epithelial *Bmal1* deletion, establishing that a functional host intestinal clock is required to translate restored metabolite rhythmicity into anti-inflammatory outcomes [38]. This finding has important translational implications: TRF interventions are expected to be most effective in individuals whose core intestinal clock machinery is intact, suggesting that chronobiotic efficacy assessment should incorporate clock gene expression status as a stratification variable.

Human evidence for the TRF-mediated restoration of microbial metabolite rhythmicity remains limited but directionally consistent with preclinical findings. Studies of time-restricted eating in metabolically at-risk populations demonstrate improvements in circulating SCFA-related metabolites and bile acid profiles, along with reductions in systemic inflammatory markers. However, the temporal resolution of metabolomics sampling in most clinical trials is insufficient to confirm the restoration of diurnal rhythmicity [78,80]. The design of TRF clinical trials specifically powered to detect changes in metabolite rhythmicity amplitude, aside from the mean concentration, represents a critical methodological priority for the field.

### 5.2. Prebiotic and Dietary Fiber Interventions

Dietary fiber supplementation augments the substrate supply available to SCFA- and indole-producing microbial taxa, amplifying the metabolite signal produced during the feeding window of the active phase of the host. Fermentable fibers such as inulin, fructooligosaccharides, and resistant starch selectively enrich butyrate-producing *Lachnospiraceae* and *Ruminococcaceae*; this selective enrichment enhances the diurnal amplitude of SCFA production when aligned with circadian-appropriate feeding timing [16,45]. The interaction between dietary fiber type, feeding timing, and microbial community composition determines the magnitude of metabolite rhythmicity augmentation, such that identical fiber doses consumed at circadian-misaligned times produce attenuated effects on metabolite rhythm amplitude relative to active-phase consumption [24]. Similarly, tryptophan-rich diets enhance substrate availability for indole derivative production, with demonstrated improvements in AhR activation and barrier function in preclinical colitis models [50,70]. These diet–metabolite–clock interactions indicate that dietary composition and timing are complementary rather than independent determinants of metabolite rhythmicity.

### 5.3. Probiotic and Postbiotic Approaches

Probiotic strains with a demonstrated capacity to produce circadian-relevant metabolites or modulate the compositional balance of metabolite-producing microbial communities represent an emerging chronobiotic strategy [57]. *Lactobacillus* and *Bifidobacterium* species contribute to SCFA production and tryptophan-to-indole catabolism; specific strains, including *Lactiplantibacillus plantarum*, have been shown to produce indole-3-lactic acid and indole-3-carboxaldehyde via AhR-activating pathways that reinforce epithelial barrier function [50]. The chronobiotic potential of probiotics lies in the metabolites they produce and their capacity to stabilize the diurnal oscillations of co-existing microbial taxa, reducing the amplitude attenuation associated with dysbiosis-driven metabolite arrhythmicity [19,81]. Postbiotic preparations, including standardized SCFA formulations and indole derivative concentrates, offer a pharmacologically precise approach to restore metabolite rhythmicity in clinical settings where probiotic engraftment is unreliable. However, their optimal time-of-administration dependence requires further systematic characterization.

Despite the mechanistic coherence of this framework, key areas of translational evidence require qualification. Clinical shift work studies are largely cross-sectional, probiotic trials report mean metabolite changes rather than rhythmicity restoration, and TRF evidence in humans lacks the temporal resolution needed to confirm diurnal amplitude recovery. Longitudinal designs with high-frequency metabolomics sampling are needed to close these gaps. The absence of standardized methods for quantifying microbial metabolite rhythmicity in humans is a key translational gap across all intervention strategies. Validated biomarkers of metabolite rhythmicity amplitude that are suitable for use in clinical trials with feasible sampling protocols would substantially advance our current capacity to evaluate chronobiotic interventions and identify responder subgroups. Furthermore, the integration of temporal metabolomics with continuous glucose monitoring and wearable circadian phase assessment represents a promising direction for next-generation chronobiotic trial design.

## 6. Conclusions

This review constructs a mechanistic framework for the circadian governance of intestinal epithelial homeostasis through microbial metabolite diversity. SCFAs and secondary bile acids are supported by the most direct experimental evidence linking rhythmic production, circadian-gated receptor signaling, and epithelial outcomes; indole derivatives represent an intermediate tier in which circadian gating of AhR is established but rhythmicity-dependent epithelial evidence remains incomplete; and BCFAs are best understood as emerging markers of host–microbiota clock coupling whose direct epithelial roles require further characterization. Their simultaneous disruption under circadian misalignment produces compound dysfunction exceeding what any single metabolite deficit can generate. Key questions remain unresolved: the circadian phases at which each metabolite class exerts maximal epithelial effects have not been systematically mapped, the relative contributions of metabolite quantity versus rhythmicity amplitude to homeostatic outcomes require time-series dissection, and clinical validation of metabolite rhythmicity restoration as a chronobiotic endpoint remains an urgent priority. Addressing these gaps is essential for translating the principles established here into evidence-based temporal nutrition strategies.

## Figures and Tables

**Table 1:** Comparative overview of major gut microbial metabolite classes in circadian intestinal homeostasis.

Category/Characteristics	Short-Chain Fatty Acids (SCFAs)	Secondary Bile Acids	Indole Derivatives	Branched-Chain Fatty Acids (BCFAs)
**I. Production and regulation**				
Principal metabolites	Acetate, propionate, butyrate	DCA, LCA	IAA, IPA, IAld, ILA, indole	Isobutyrate, isovalerate
Primary substrate	Dietary fiber	Host primary BAs (CA, CDCA)	Dietary tryptophan (Trp)	BCAAs (Leu, Ile, Val)
Key producing taxa	*Lachnospiraceae*, *Ruminococcaceae*	BSH^+^ Clostridiales, *Bacteroides*	*Lactobacillus*, *Bifidobacterium*	Protein-fermenting anaerobes
Peak phase	Dark/active phase (ZT12–16) [15,16]	Diurnal (13:00, 21:00 in humans) [17]	Active feeding phase [18]	Parallel to SCFA rhythms [7]
Primary entrainment cue	Feeding-fasting + intestinal clock [1,7]	Hepatic CYP7A1 + microbial BSH [19,20]	Dietary Trp + microbial oscillation [18,21]	Host intestinal clock (*Bmal1*-IEC) [7]
Clock-disruption effect	Amplitude ↓; fermenting taxa arrhythmia [22,23]	Pool rhythmicity lost (sleep deprivation) [17,24]	Indole/IAA production ↓ [21]	BCFA amplitude ↓ (*Bmal1*-IEC KO) [7]
**II. Signaling and barrier function**				
Principal receptors (IEC)	FFAR2/3, GPR109A, HDAC3	FXR (nuclear), TGR5 (membrane)	AhR, PXR	FFAR2/3 (inferred); HDACs (inferred, lower potency)
Intracellular mechanism	HDAC inhibition → H3K18ac/H4K12bu ↑ [8,25]	FXR–RXRα; cAMP/PKA → MLCK [26,27]	AhR–ARNT → XRE; AhR–BMAL1 competition at *Per1* [28]	HDAC inhibition (lower potency, inferred from structural analogy with SCFAs) [25,29]
Tight junction targets	↑ Claudin-1/-7/-15, Occludin, ZO-1 [30,31]	↑ Occludin, Claudin (MLCK-mediated) [26,27]	↑ Claudin-1, Occludin, ZO-1 (AhR) [32,33]	Not directly demonstrated (N.D.)
Mucus barrier effects	↑ MUC2 rhythmicity (HDAC3-dependent) [7]	↑ Goblet cell differentiation (FXR) [26]	↑ MUC2, MUC4, TFF3 (AhR/PXR) [34,35]	Not determined (N.D.)
**III. Immunity and renewal**				
Immune targets/mediators	Tregs (Foxp3); tuft cells (HDAC3) [36,37]	Innate immune suppression (FXR) [26,38]	ILC3 → IL-22; Tregs (AhR) [28,32,33]	Not determined (N.D.)
Immune rhythm loss effect	↓ Mucosal tolerance; type 2 dysregulation [36]	↑ Colitis susceptibility (*Bmal1* KO) [39]	↓ IL-22 amplitude; ↓ defense [21]	Lymphoid organ weight ↑ (*Bmal1*-IEC KO; mechanism unclear) [7]
Epithelial renewal	Phase-dependent differentiation; stem cell suppression [40,41]	TGR5 → Cyclin D1 → proliferation [27]	IL-22-driven regeneration; mucus renewal [35,42]	Rhythmicity serves as host–microbiota clock coupling readout; direct renewal effects not established [7]
**IV. Clock feedback and intervention**				
Metabolite → clock feedback	HDAC inhibition → *Per1/2* ↑; GLP-1 → PER2 phase advance [8,16,43]	LCA → CRY2 stabilization (period lengthening) [44]	AhR–BMAL1 competition at the *Per1* promoter [28]	Inferred from SCFA analogy; not directly demonstrated (N.D.)
TRF intervention effect	Restores SCFA rhythm (*Bmal1*-IEC dependent) [45,46]	Restores BA profiles and immunity [45]	↑ Trp-metabolizing taxa rhythms [47]	Not tested (N.T.)
Nutritional/postbiotic strategy	Fermentable fiber; sodium butyrate [16,19]	BSH-active probiotics [48]	Trp-rich diet; *L. plantarum* [49,50]	BCAA restriction (putative; not tested) (N.T.)
Translational gap	Amplitude vs. total quantity; sampling frequency [51]	Human in vivo BA temporal resolution [17]	Phase-optimal ligand delivery timing [18]	Direct epithelial effects and clinical biomarker potential not validated

Abbreviations: AhR, aryl hydrocarbon receptor; BA, bile acid; BCAA, branched-chain amino acid; BCFA, branched-chain fatty acid; BSH, bile salt hydrolase; FXR, farnesoid X receptor; HDAC, histone deacetylase; IEC, intestinal epithelial cell; ILC3, group 3 innate lymphoid cell; MLCK, myosin light chain kinase; N.D., not determined; N.T., not tested; PXR, pregnane X receptor; SCFA, short-chain fatty acid; TGR5, Takeda G protein-coupled receptor 5; TRF, time-restricted feeding; ZT, Zeitgeber time; ↑, increased or upregulated; ↓, decreased or downregulated; →, leads to or is associated with.

## Data Availability

No new data were created or analyzed in this study. Data sharing is not applicable to this paper.

## Abbreviations

The following abbreviations are used in this manuscript:

AhR	Aryl hydrocarbon receptor
BCFA	Branched-chain fatty acid
BSH	Bile salt hydrolase
DCA	Deoxycholic acid
HDAC	Histone deacetylase
IAA	indole-3-acetic acid
IAld	indole-3-aldehyde
ILA	indole-3-lactic acid
IPA	indole-3-propionic acid
LCA	Lithocholic acid
PXR	Pregnane X receptor
SCFA	Short-chain fatty acid
SCN	Suprachiasmatic nucleus
TRF	Time-restricted feeding
ZT	Zeitgeber time
